# Supplementary material for: Unique functions for Notch4 in murine embryonic lymphangiogenesis
Source: Angiogenesis. 2021 Oct 19;25(2):205–24. doi: 10.1007/s10456-021-09822-5 (PMC9054879; doi:10.1007/s10456-021-09822-5)
Supplement: Supplementary file 1 — Supplementary file1 (PDF 11764 kb) [file 10456_2021_9822_MOESM1_ESM.pdf]

Supplemental Files:

Article Title: *Unique functions for Notch4 in murine embryonic lymphangiogenesis*

Journal Name: *Angiogenesis*

Authors and Affiliations: Ajit Muley[1]*, Minji Kim Uh[1,2]*, Glicella Salazar-De Simone[1], Bhairavi Swaminathan[3], Jennifer M. James[4], Aino Murtomaki[1,5,6], Seock Won Youn[3], Joseph D. McCarron[1], Chris Kitajewski[1], Maria Gnarra Buethe[1], Gloria Riitano[1,7], Yoh-suke Mukouyama[4], Jan Kitajewski[3], Carrie J. Shawber[1,8]

[1]Department of Obstetrics and Gynecology, Columbia University Medical Center, New York, NY 10032, USA.

[2]Department of Pharmacology, Columbia University Medical Center, New York, NY 10032, USA.

[3]Department of Physiology and Biophysics, University of Illinois Chicago, Chicago, IL 60612, USA.

[4]Laboratory of Stem Cell and Neuro-Vascular Biology, Cell and Developmental Biology Center, National Heart, Lung, and Blood Institute, National Institutes of Health, Bethesda, MD 20892, USA.

[5]Wihuri Research Institute, Biomedicum Helsinki, Haartmaninkatu, Helsinki, 8,00290 Finland

[6]Translational Cancer Medicine Program, Faculty of Medicine and Helsinki Institute of Life Science, University of Helsinki, Helsinki, , FI-00014, Finland

[7]Departments of Molecular Medicine and Experimental Medicine, Sapienza University, Rome, 00185, Italy.

[8]Department of Surgery, Columbia University Medical Center, New York, NY 10032, USA.

*These first authors contributed equally to this work

Corresponding Author:

Carrie J. Shawber

cjs2002@cumc.columbia.edu

Supplemental Tables 1-3

Supplemental Table 1. Primers for quantitative RT-PCR using gene specific standards

Gene	Upper Primer	Lower Primer
β-actin	5' CGAGGCCCAGAGCAAGAGAG 3'	5' CTCGTAGATGGGCACAGTGTG 3'
Dll4	5' CGGGTCATCTGCAGTGACAAC 3'	5' AGTTGAGATCTTGGTCACAAAACAG 3'
Hes1	5' CCCAACGCAGTGTCACCTTC 3'	5' TACAAAGGCGCAATCCAATATG 3'
Hey1	5' ACGAGAATGGAAACTTGAGTT C 3'	5' AACTCCGATAGTCCATAGCAAG 3'
Hey2	5' ATGAGCATAGGATTCCGAGAGTG 3'	5' GGCAGGAGGCACTTCTGAAG 3'
Notch1	5' CTCACCTGGTGCAGACCCAG 3'	5' GCACCTGTAGCTGGTGGCTG 3'
Notch4	5' GGTGACACCCCTGATGTCAG 3'	5' AGCCTGGCAGCCAGCATC 3'
Lyve1	5′ TAGCTTTGAAACTTGCAGCTATG 3'	5′ TCAACAAATGGTTCAGTTTCTGTAG 3'
Podoplanin	5′ CCCAGGAGAGCAACAACTCAAC 3'	5′ CTCGATGCGAATGCCTGTTAC 3'
Prox1	5′ ACGTAAAGTTCAACAGATGCATTAC 3'	5′ CCAGCTTGCAGATGACCTTG 3'
Vegfr2	5′ GGACTGGCTTTGGCCCAAT 3′	5′ CTTGCTGTCCCAGGAAATTCTG 3′
Vegfr3	5′ GAGACCTGGCTGCTCGGAAC 3'	5′ TCAGCATGATGCGGCGTATG 3'

Supplemental Table 2. Quantitative RT-PCR primers for mRNA seq validation

Gene	Forward Primer	Reverse Primer
Ackr3	5' CCAAGACCACAGGCTATGACA '3	5' TGGTTGTGCTGCACGAGACTGA '3
β-actin	5' CGAGGCCCAGAGCAAGAGAG '3	5' CTCGTAGATGGGCACAGTGTG '3
Bmp2	5' TTCGGCCTGAAACAGAGACC '3	5' CCTGAGTGCCTGCGATACAG '3
Ccl2	5' AGAATCACCAGCAGCAAGTGTCC '3	5' TCCTGAACCCACTTCTGCTTGG '3
Cxcr4	5' ACGCCACCAACAGTCAGAG '3	5' AGTCGGGAATAGTCAGCAGGA '3
Efnb2	5' TATGCAGAACTGCGATTTCCAA '3	5' TGGGTATAGTACCAGTCCTTGTC '3
Inhbb	5' CGGGTCCGCCTATACTTCTTC '3	5' CGTAGGGCAGGAGTTTCAGG '3
Hes1	5' CCTGTCATCCCCGTCTACAC '3	5' CACATGGAGTCCGCCGTAA '3
Hes4	5' GAGCGCGTATTAACGAGAGCCT '3	5' CTCACGGTCATCTCCAGGATGT '3
Hes5	5' CTCAGCCCCAAAGAGAAAAA '3	5' GACAGCCATCTCCAGGATGT '3
Hey1	5' ATCTGCTAAGCTAGAAAAAGCCG '3	5' GTGCGCGTCAAAGTAACCT '3
Hey2	5' GCCCGCCCTTGTCAGTATC '3	5' CCAGGGTCGGTAAGGTTTATTG '3
HeyL	5' GGCTGCTTACGTGGCTGTT '3	5' GACCCAGGAGTGGTAGAGCAT '3
Sema3g	5' CAGAGGATGGGACCTACGATG '3	5' GTTGGCACCTTAAACACCTGG '3
Tgfrb2	5' GTAGCTCTGATGAGTGCAATGAC '3	5' CAGATATGGCAACTCCCAGTG '3
Unk5b	5' CTGGGACCTTATGCCTTCAA '3	5' CGCTTTGGTGGCAAAGTAAT '3

Supplemental Table 3. Antibodies

Antigen	Supplier	Catologue #	Use
β-ACTIN	Abclonal	AC038	Westerns
CD31	Pharmingen	553370	wholemount IHC
DLL4	R&D Systems	AF1389	wholemount IHC
JAG1	R&D Systems	AF599	wholemount IHC
KI67	Abcam	ab15580	wholemount IHC
LYVE1	Abcam	ab14917	wholemount, section IHC
LYVE1	Ebiosciences	14-0443	wholemount IHC
PROX1	Angiobio	11-002	wholemount IHC
NOTCH1	R&D Systems	AF1057	section IHC
NOTCH1	Cell Signaling	3608S	wholemount IHC
NOTCH4	J. Kitajewski	RB2-2	section IHC, Westerns
NOTCH4	BioXCell	BE0129	wholemount IHC
phospho-AKT	Cell Signaling	13038	Cell IHC
phospho-ERK	Cell Signaling	4370	Cell IHC
VEGFR3	R&D Systems	AF743	wholemount IHC

Supplemental Figures 1-9

Fig. S1 Murine dermal lymphangiogenesis and NOTCH1 and NOTCH4 expression. **a**) The lymphangiogenic plexus consists of a maturing plexus (1) and active lymphangiogenic fronts (2) which migrates towards the lateral midline (3, dashed line). LYVE1 and CD31 staining of E14.5 dorsal skin. Scale bar, 100µm. **b, c**) Cross-sections of wild-type dermis stained for LYVE1 and **b**) NOTCH1 or **c**) NOTCH4. White arrowheads mark LYVE1+/NOTCH1+ and LYVE1+/NOTCH4+ LECs. Yellow arrowheads mark NOTCH1+ blood vessels. Blue arrowheads mark LYVE1+/NOTCH4+ macrophages. Scale bars, 50µm.

Fig. S2 Dll4 and Jag1 activated Notch signaling in HdLECs. **a**) Quantification of ectopic *Dll4* and *Jag1* transcript levels in HeLa cells expressing GFP, DLL4, JAG1 or both DLL4 and JAG1. *Dll4* and *Jag1* qRT-PCR of HeLa cell lines used in co-culture. Data presented as relative transcript levels ± s.e.m. **b**) Notch/CSL luciferase reporter assay of ligand-expressing or GFP-expressing HeLa cells (y-axis) co-cultured with HdLEC. Data presented as fold luciferase induction over HdLEC co-cultured with GFP-HeLa controls ± s.e.m. Representative experiments presented and performed in triplicate. * $p < 0.02$, ** $p < 0.001$. ns – not significant.

Fig. S3 Effect of DLL4/Notch signaling on Notch pathway and lymphangiogenic genes in HdLECs. **a**) Heatmap of Notch pathway genes. **b**) Heatmap of lymphangiogenic genes.

Fig. S4 VEGF and Notch signaling in HUVEC and HdLECs. **a**) *Notch1*, *Notch4*, and *Dll4* qRT-PCR of HUVEC treated for 5 hours with either VEGF-A or VEGF-C. Data presented as fold induction over HUVEC in no growth factors ± s.d. * $p < 0.05$. b) *Vegfr2*, *Vegfr3*, *Prox1*, *Podoplanin*, and *Lyve1* qRT-PCR of HdLEC expressing GFP, N1IC, or N4/Int-3. Data presented as fold induction over GFP expressing HdLECs ± s.e.m. Mean for > three experiments presented. T-Test * $p < 0.05$.

Fig. S5 Validation of the *Notch4* null mice. **a**) P4 dorsal dermal tissue cross-sections from *Notch4*$^{-/-}$ and wild-type littermates were stained for VEGFR3 and NOTCH4. NOTCH4 expression was absent in the lymphatic endothelium (white arrowheads) and epithelial cells of the hair follicle (yellow arrowheads) and dermis in *Notch4*$^{-/-}$ tissues. **b**) Representative Western blot of E14.5 *Notch4*$^{-/-}$ and wild-type tissues probed with antibodies against the cytoplasmic domain of NOTCH4 (N4IC) or βACTIN. **c**) Mean N4IC expression normalized by βACTIN for wild-type (n=3) and *Notch4*$^{-/-}$ (n=4) Westerns determined by densitometry. Data presented ± s.e.m. T-test: * $p < 0.05$. **d**) P4 dorsal dermal tissue cross-sections from *Notch4*$^{-/-}$ and wild-type littermates were stained for VEGFR3 and NOTCH1. NOTCH1 expression was unchanged in the VEGFR3+ lymphatics (white arrowheads). Scale bars, 50µm.

Fig. S6 Lymphatic branching, proliferation and canonical Notch signaling was unaffected in E14.5 *Notch4*$^{-/-}$ dermis. **a)** Quantification of the average number of branch-points normalized to unit of vessel length. Data presented ± sem. wt (n=7), *N4*$^{+/-}$ (n=13), *N4*$^{-/-}$ (n=7). **b**) LYVE1 and KI67 staining of E14.5 *Notch4*$^{-/-}$ and wild-type dermis. Scale bars, 100µm. **c**) Quantification of PROX1+/KI67+ LECs normalized to lymphatic vascular area ± s.e.m. wt (n=3), *N4*$^{-/-}$ (n=4) **d**) Number of PROX1+ and PROX1+/NVR+ LECs was determined for individual sprout at the lymphangiogenic front until the first branch-point for E14.5 *ProxTOM;NVR* and *Notch4*$^{-/-}$*;ProxTOM;NVR* wholemounts stained for LYVE1, Data presented as percent PROX1+/NVR+ LECs relative to total Prox1+ LECs ± s.e.m. wt (n=6), *N4*$^{-/-}$ (n=3)

Fig. S7 Dermal blood vasculature was unaffected in *Notch4*$^{-/-}$ mice. **a**) CD31 staining of E14.5 dorsal dermal wholemounts from *N4*$^{-/-}$ and control *N4*$^{+/-}$ littermates. **b**) Quantification of average CD31 intensity normalized by area. Data presented ± s.e.m. *N4*$^{+/-}$ (n=3), *N4*$^{-/-}$ (n=4). **c**) Quantification of the average number of branch-points per field of view. Data presented ± sem. *N4*$^{+/-}$ (n=3), *N4*$^{-/-}$ (n=5).

Fig. S8 Increase in blunt-ended lymphatic sprouts in E14.5 *Notch4*$^{-/-}$ dermis. **a)** High magnification images of LYVE1+ sprouts at the lymphangiogenic front. White arrowheads mark filopodia extending from a spiky-ended sprouts. Red arrowheads mark blunt-ended sprouts, Scale bars, 20µm. **b)** Quantification of the sprout morphology at the lymphangiogenic front. Blunt-ended sprouts are rounded with reduced filopodia. Spiky-ended sprouts have multiple filopodia. Data presented as percent blunt or spiky phenotype relative to total sprouts ± s.e.m. One-way ANOVA: $p = 0.004$, T-Test: *$p < 0.003$, wt (n=5), *N4*$^{+/-}$ (n=7), *N4*$^{-/-}$ (n=5).

Fig. S9 Dermal blood vasculature was unaffected in mice with LEC loss of canonical Notch signaling**.** *Prox1CreER*^{T2} and *DNMAML*$^{fl/fl}$ mice were crossed and tamoxifen administered at E12.5 and dorsal dermis analyzed at E14.5. **a**) CD31 staining of *Prox1CreER*^{T2}*;DNMAML*$^{fl/+}$ (*DNMAML*^{LEC}) mutant and *DNMAML*$^{fl/+}$ (control) dermis. White dashed line denotes the midline. Scale bars, 1000µm. **b**) Quantification of average CD31 intensity normalized by area. Data presented ± s.e.m. control (n=3), *DNMAML*^{LEC} (n=6).

Muley/Kim et al. Supp. Figure 1

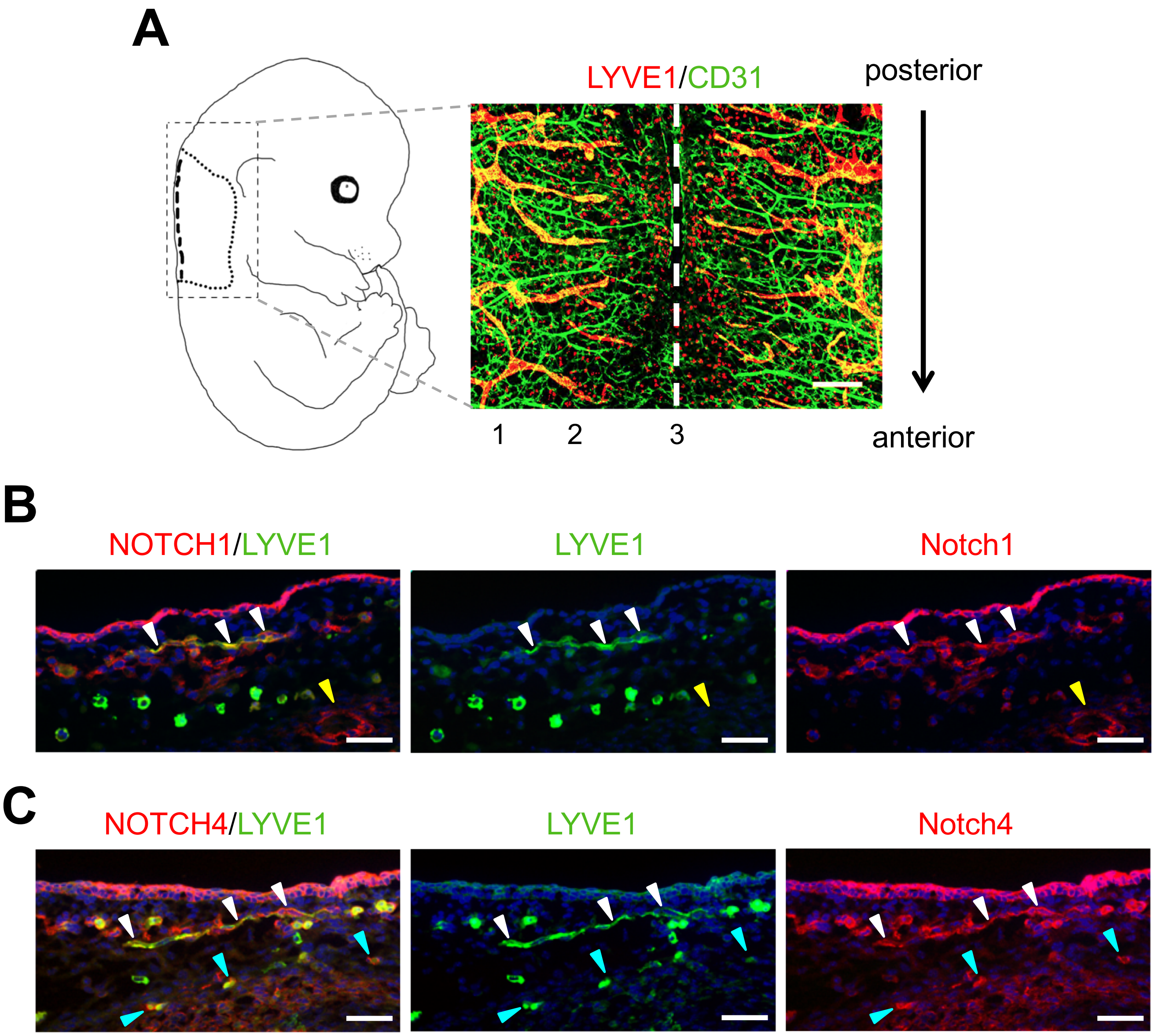

Fig. S1 Murine dermal lymphangiogenesis and NOTCH1 and NOTCH4 expression. **a**) The lymphangiogenic plexus consists of a maturing plexus (1) and active lymphangiogenic fronts (2) which migrates towards the lateral midline (3, dashed line). LYVE1 and CD31 staining of E14.5 dorsal skin. Scale bar, 100µm. **b, c**) Cross-sections of wild-type dermis stained for LYVE1 and **b**) NOTCH1 or **c**) NOTCH4. White arrowheads mark LYVE1+/NOTCH1+ and LYVE1+/NOTCH4+ LECs. Yellow arrowheads mark NOTCH1+ blood vessels. Blue arrowheads mark LYVE1+/NOTCH4+ macrophages. Scale bars, 50µm.

Muley/Kim et al., Supp. Figure 2

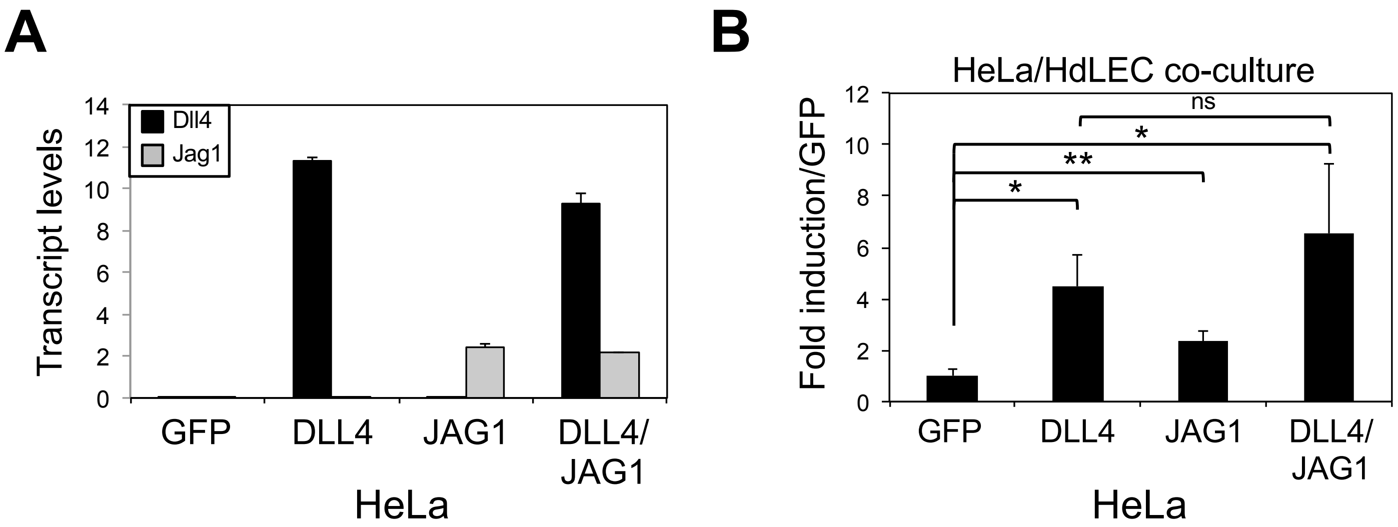

Fig. S2 Dll4 and Jag1 activated Notch signaling in HdLECs. **a**) Quantification of ectopic *Dll4* and *Jag1* transcript levels in HeLa cells expressing GFP, DLL4, JAG1 or both DLL4 and JAG1. *Dll4* and *Jag1* qRT-PCR of HeLa cell lines used in co-culture. Data presented as relative transcript levels ± s.e.m. **b**) Notch/CSL luciferase reporter assay of ligand-expressing or GFP-expressing HeLa cells (y-axis) co-cultured with HdLEC. Data presented as fold luciferase induction over HdLEC co-cultured with GFP-HeLa controls ± s.e.m. Representative experiments presented and performed in triplicate. * $p < 0.02$, ** $p < 0.001$. ns – not significant.

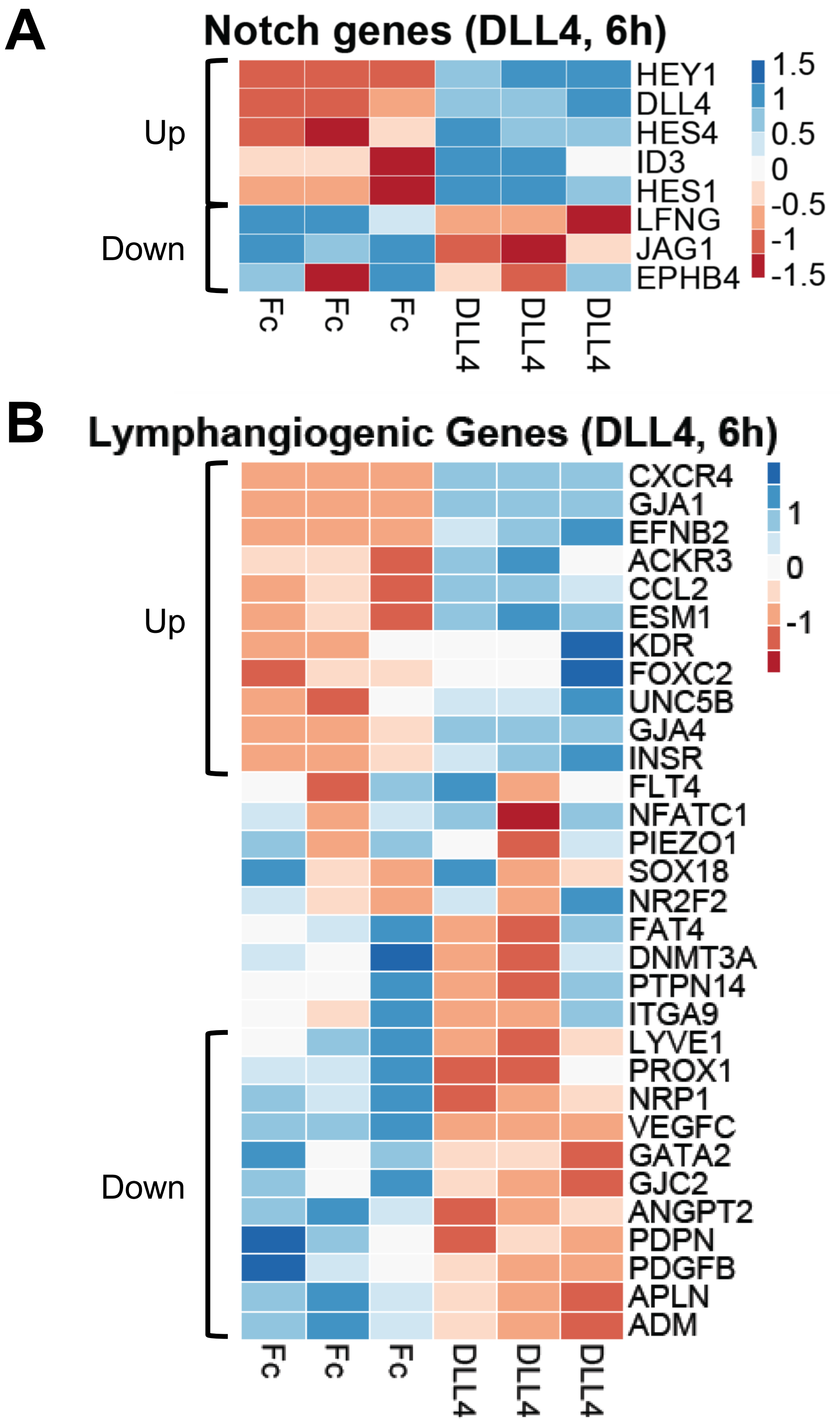

Fig. S3 Effect of DLL4/Notch signaling on Notch pathway and lymphangiogenic genes in HdLECs. **a**) Heatmap of Notch pathway genes. **b**) Heatmap of lymphangiogenic genes.

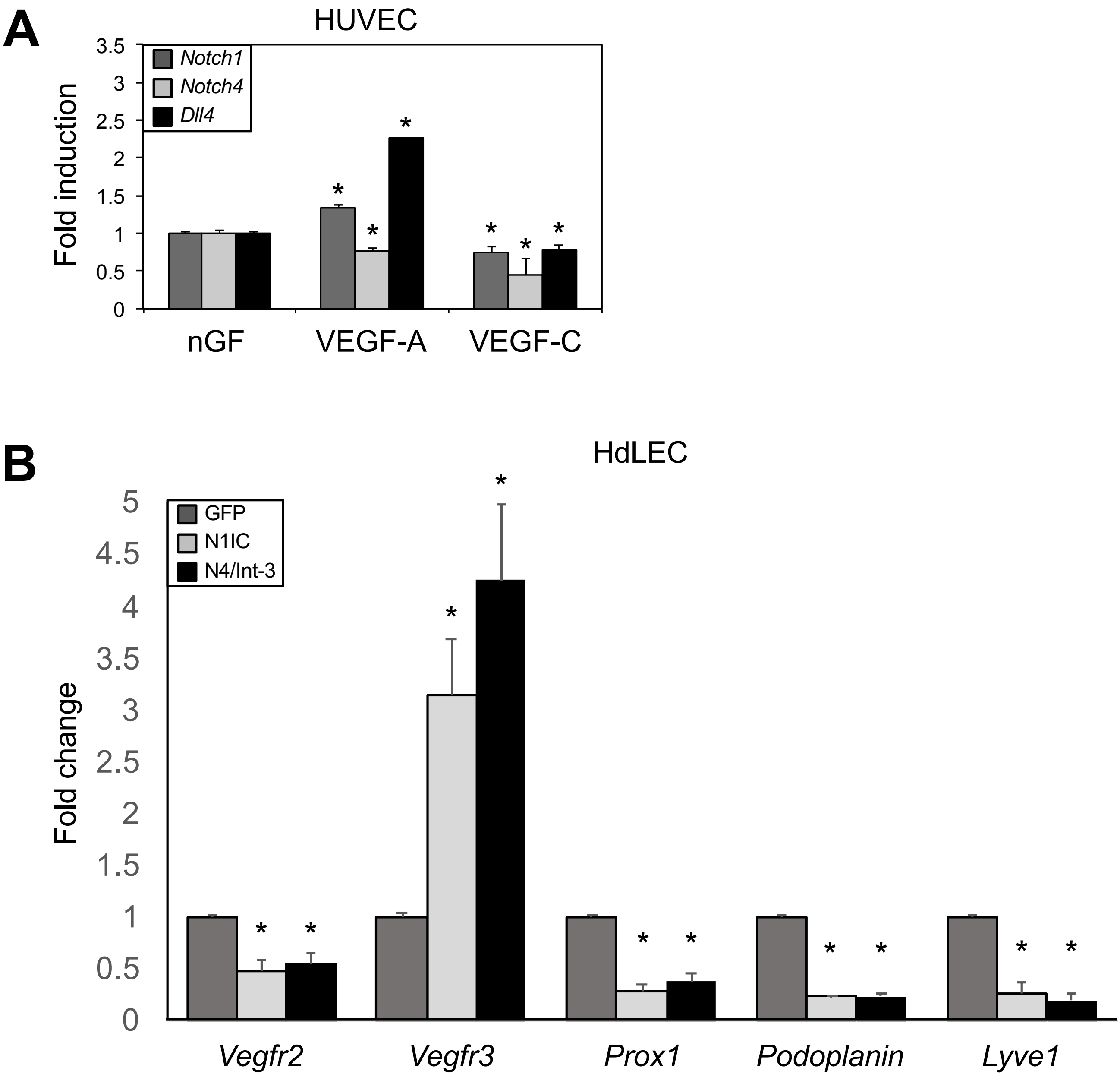

Fig. S4 VEGF and Notch signaling in HUVEC and HdLECs. **a**) *Notch1*, *Notch4*, and *Dll4* qRT-PCR of HUVEC treated for 5 hours with either VEGF-A or VEGF-C. Data presented as fold induction over HUVEC in no growth factors ± s.d. * p< 0.05. b) *Vegfr2*, *Vegfr3*, *Prox1*, *Podoplanin*, and *Lyve1* qRT-PCR of HdLEC expressing GFP, N1IC, or N4/Int-3. Data presented as fold induction over GFP expressing HdLECs ± s.e.m. Mean for > three experiments presented. T-Test * p< 0.05.

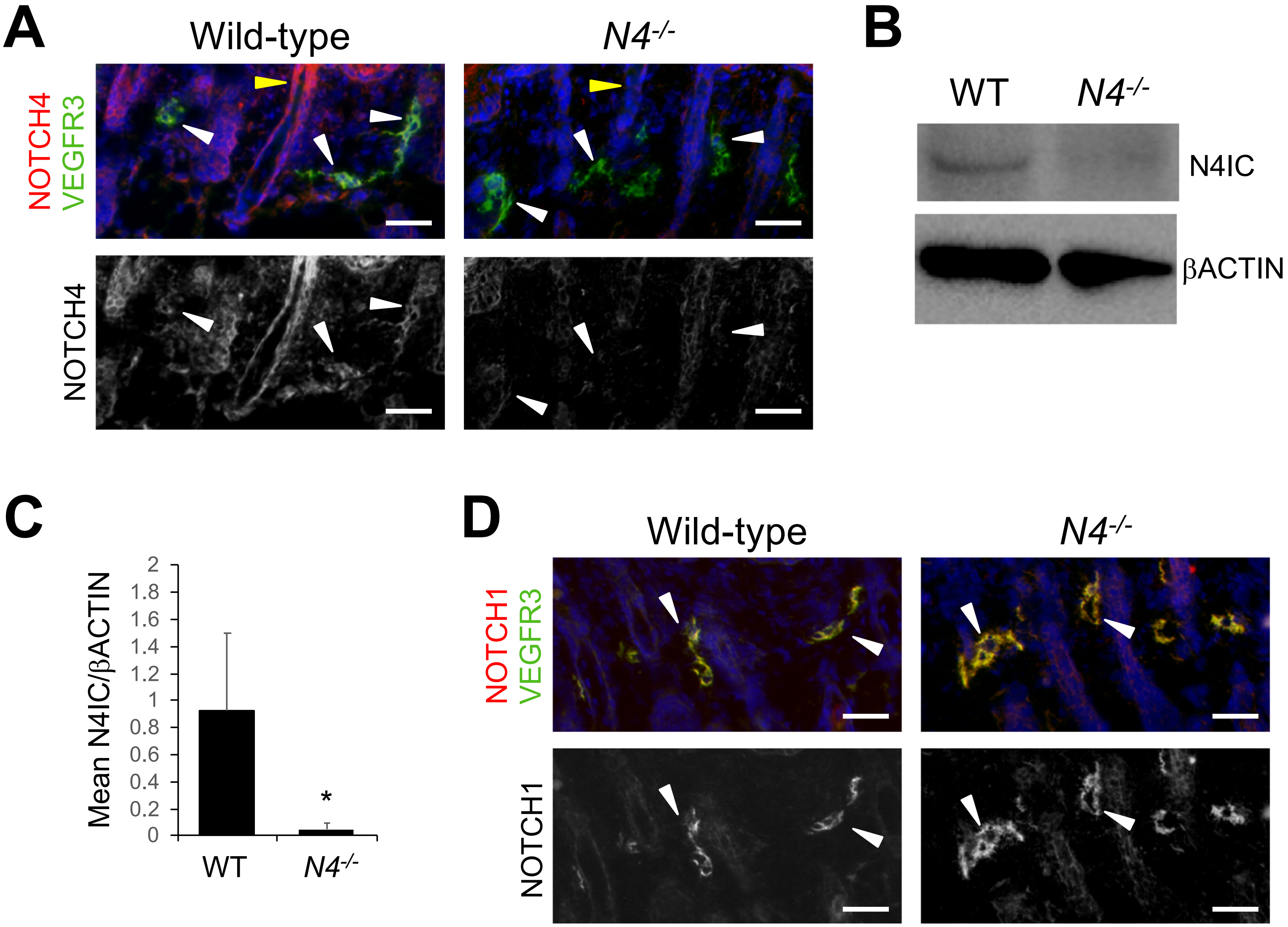

Fig. S5 Validation of the *Notch4* null mice. **a**) P4 dorsal dermal tissue cross-sections from *Notch4*$^{-/-}$ and wild-type littermates were stained for VEGFR3 and NOTCH4. NOTCH4 expression was absent in the lymphatic endothelium (white arrowheads) and epithelial cells of the hair follicle (yellow arrowheads) and dermis in *Notch4*$^{-/-}$ tissues. **b**) Representative Western blot of E14.5 *Notch4*$^{-/-}$ and wild-type tissues probed with antibodies against the cytoplasmic domain of NOTCH4 (N4IC) or βACTIN. **c)** Mean N4IC expression normalized by βACTIN for wild-type (n=3) and *Notch4*$^{-/-}$ (n=4) Westerns determined by densitometry. Data presented ± s.e.m. T-test: * $p < 0.05$. **d**) P4 dorsal dermal tissue cross-sections from *Notch4*$^{-/-}$ and wild-type littermates were stained for VEGFR3 and NOTCH1. NOTCH1 expression was unchanged in the VEGFR3+ lymphatics (white arrowheads). Scale bars, 50µm.

Muley/Kim et al. Supp. Figure 6

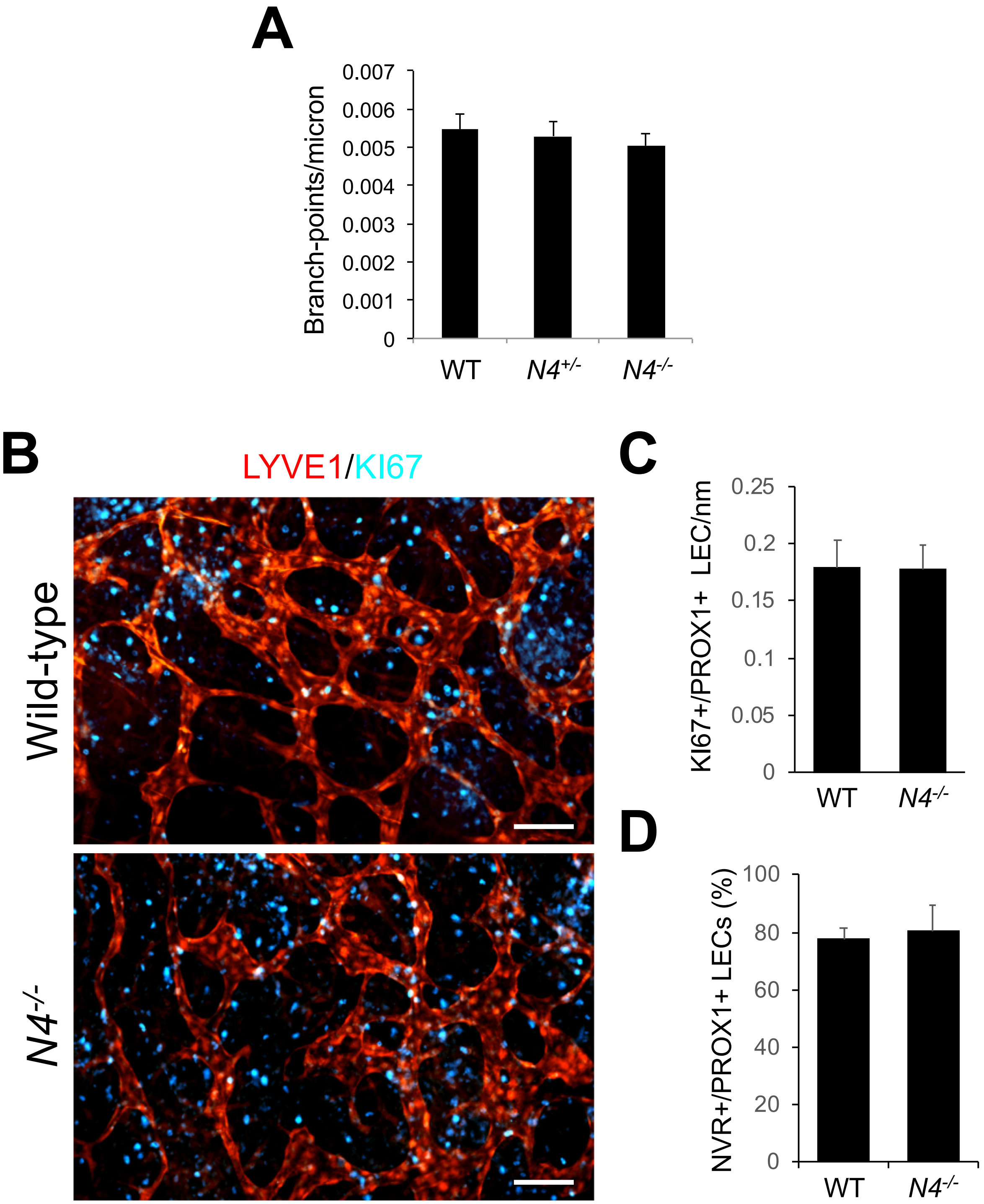

Fig. S6 Lymphatic branching, proliferation and canonical Notch signaling was unaffected in E14.5 *Notch4*$^{-/-}$ dermis. **a)** Quantification of the average number of branch-points normalized to unit of vessel length. Data presented ± sem. wt (n=7), *N4*$^{+/-}$ (n=13), *N4*$^{-/-}$ (n=7). **b**) LYVE1 and KI67 staining of E14.5 *Notch4*$^{-/-}$ and wild-type dermis. Scale bars, 100μm. **c**) Quantification of PROX1+/KI67+ LECs normalized to lymphatic vascular area ± s.e.m. wt (n=3), *N4*$^{-/-}$ (n=4) **d**) Number of PROX1+ and PROX1+/NVR+ LECs was determined for individual sprout at the lymphangiogenic front until the first branch-point for E14.5 *ProxTOM;NVR* and *Notch4*$^{-/-}$*;ProxTOM;NVR* wholemounts stained for LYVE1, Data presented as percent PROX1+/NVR+ LECs relative to total Prox1+ LECs ± s.e.m. wt (n=6), *N4*$^{-/-}$ (n=3)

Muley/Kim et al., Supp. Figure 7

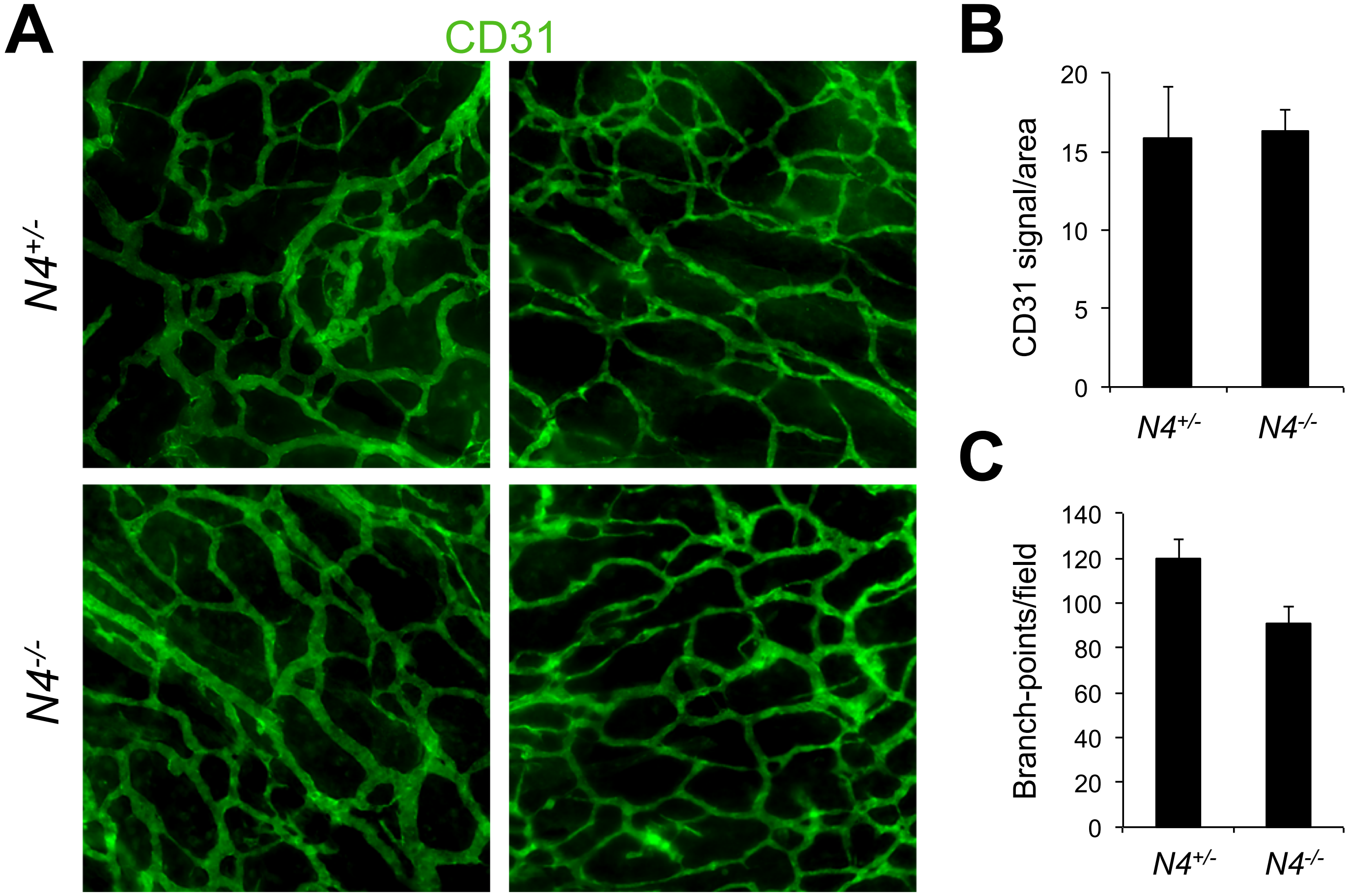

Fig. S7 Dermal blood vasculature was unaffected in *Notch4*$^{-/-}$ mice. **a**) CD31 staining of E14.5 dorsal dermal wholemounts from *N4*$^{-/-}$ and control *N4*$^{+/-}$ littermates. **b**) Quantification of average CD31 intensity normalized by area. Data presented ± s.e.m. *N4*$^{+/-}$ (n=3), *N4*$^{-/-}$ (n=4). **c**) Quantification of the average number of branch-points per field of view. Data presented ± sem. *N4*$^{+/-}$ (n=3), *N4*$^{-/-}$ (n=5).

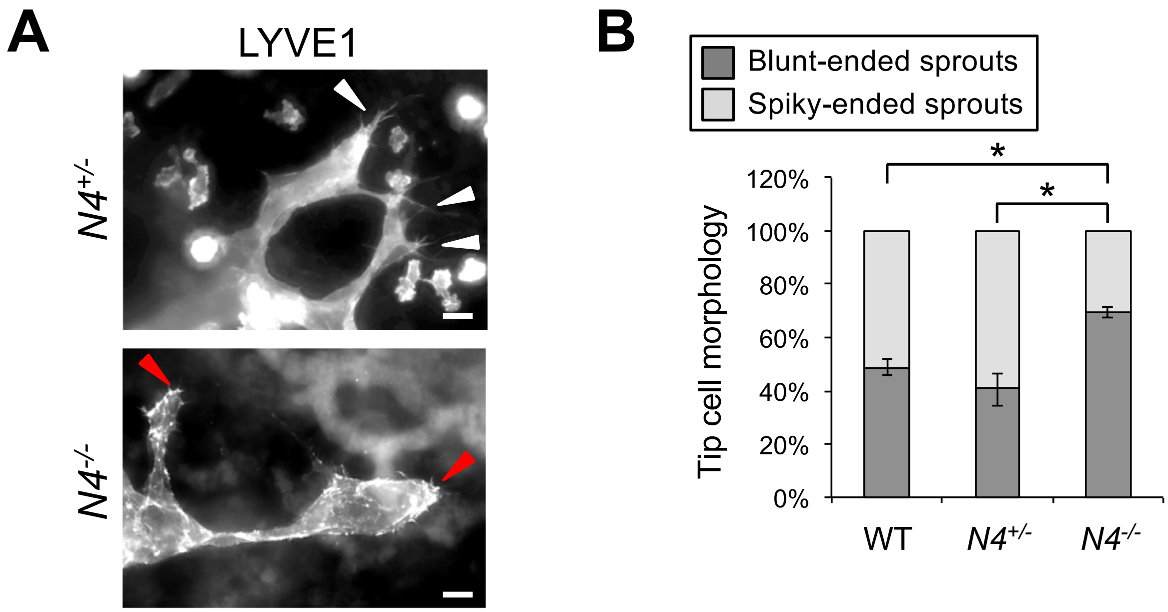

Fig. S8 Increase in blunt-ended lymphatic sprouts in E14.5 *Notch4*$^{-/-}$ dermis. **a)** High magnification images of LYVE1+ sprouts at the lymphangiogenic front. White arrowheads mark filopodia extending from a spiky-ended sprouts. Red arrowheads mark blunt-ended sprouts, Scale bars, 20µm. **b)** Quantification of the sprout morphology at the lymphangiogenic front. Blunt-ended sprouts are rounded with reduced filopodia. Spiky-ended sprouts have multiple filopodia. Data presented as percent blunt or spiky phenotype relative to total sprouts ± s.e.m. One-way ANOVA: $p= 0.004$, T-Test: *$p< 0.003$, wt (n=5), *N4*$^{+/-}$ (n=7), *N4*$^{-/-}$ (n=5).

Muley/Kim et al., Supp. Figure 9

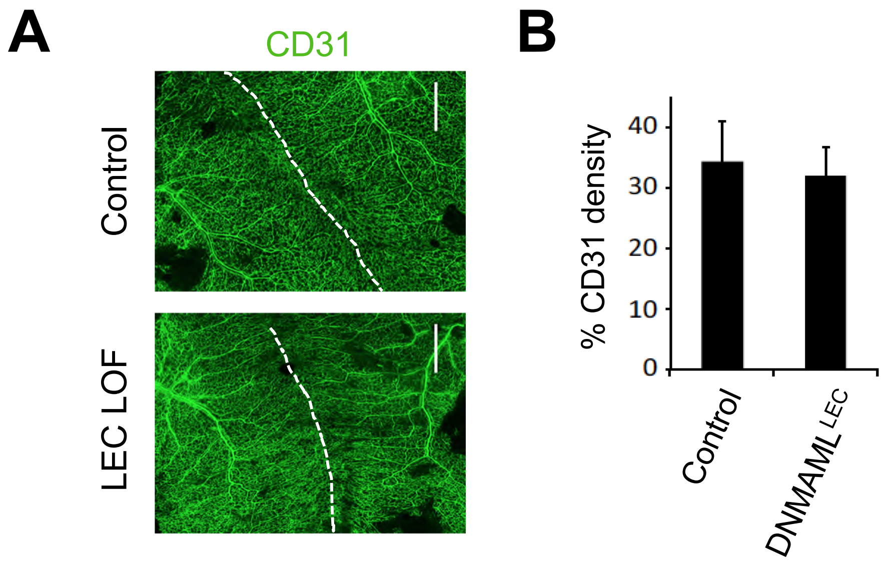

Fig. S9 Dermal blood vasculature was unaffected in mice with LEC loss of canonical Notch signaling**.** *Prox1CreER*^{T2} and *DNMAML*$^{fl/fl}$ mice were crossed and tamoxifen administered at E12.5 and dorsal dermis analyzed at E14.5. **a**) CD31 staining of *Prox1CreER*^{T2}*;DNMAML*$^{fl/+}$ (*DNMAML*^{LEC}) mutant and *DNMAML*$^{fl/+}$ (control) dermis. White dashed line denotes the midline. Scale bars, 1000µm. **b**) Quantification of average CD31 intensity normalized by area. Data presented ± s.e.m. control (n=3), *DNMAML*^{LEC} (n=6).
